# Testosterone Protects Against Severe Influenza by Reducing the Pro-Inflammatory Cytokine Response in the Murine Lung

**DOI:** 10.3389/fimmu.2020.00697

**Published:** 2020-04-22

**Authors:** Berfin Tuku, Stephanie Stanelle-Bertram, Julie Sellau, Sebastian Beck, Tian Bai, Nancy Mounogou Kouassi, Annette Preuß, Stefan Hoenow, Thomas Renné, Hanna Lotter, Gülsah Gabriel

**Affiliations:** ^1^Department Viral Zoonoses - One Health, Heinrich Pette Institute, Leibniz Institute for Experimental Virology, Hamburg, Germany; ^2^Research Group Molecular Infection Immunology, Bernhard Nocht Institute for Tropical Medicine, Hamburg, Germany; ^3^Institute for Clinical Chemistry and Laboratory Medicine, University Medical Center Hamburg-Eppendorf, Hamburg, Germany; ^4^Institute of Virology, University of Veterinary Medicine Hannover, Hanover, Germany

**Keywords:** influenza A virus, sex differences, testosterone, 2009 H1N1, androgens

## Abstract

Influenza A virus pathogenesis may differ between men and women. The 2009 H1N1 influenza pandemic resulted in more documented hospitalizations in women compared to men. In this study, we analyzed the impact of male sex hormones on pandemic 2009 H1N1 influenza A virus disease outcome. In a murine infection model, we could mimic the clinical findings with female mice undergoing severe and even fatal 2009 H1N1 influenza compared to male mice. Treatment of female mice with testosterone could rescue the majority of mice from lethal influenza. Improved disease outcome in testosterone treated female mice upon 2009 H1N1 influenza A virus infection did not affect virus titers in the lung compared to carrier-treated females. However, reduction in IL-1β cytokine expression levels strongly correlated with reduced lung damage and improved influenza disease outcome in female mice upon testosterone treatment. In contrast, influenza disease outcome was not affected between castrated male mice and non-castrated controls. Here, influenza infection resulted in reduction of testosterone expression in male mice. These findings show that testosterone has protective functions on the influenza infection course. However, 2009 H1N1 influenza viruses seem to have evolved yet unknown mechanisms to reduce testosterone expression in males. These data will support future antiviral strategies to treat influenza taking sex-dependent immunopathologies into consideration.

## Introduction

Females in their reproductive age experience more severe disease following influenza A virus infection than males (1, 2). Hospitalization rates during influenza seasons were reported to be higher in male children and in elderly males. However, during the reproductive age, females are more likely to be hospitalized than males (3). This was further supported and highlighted during the 2009 H1N1 influenza pandemic. Here, females were reported to develop more severe influenza disease compared to males in multiple published datasets (4–6). In line, females were more likely to die upon avian H7N9 or H5N1 influenza A virus infection than males (7, 8). These epidemiological observations could be mimicked in murine infection models (7). Yet, the underlying mechanisms of sex-specific influenza disease outcome are largely unknown. In males, testosterone is the predominant sex hormone (further described as “male sex hormone”), whereas in females the predominant sex hormones are estradiol and progesterone (further described as “female sex hormones”). In a murine influenza infection model, it was shown that the female sex hormones, 17β-estradiol and progesterone, have a protective effect on influenza disease outcome in females (9, 10). In this study, we analyzed the impact of the major male sex hormone testosterone on influenza disease outcome using a pre-clinical murine infection model.

## Materials and Methods

### Animal Experiments

C57Bl/6JRccHsd mice were purchased from Envigo RMS Harlan Laboratories (Rossdorf, Germany). Eight weeks old mice were anesthetized intraperitoneally with ketamine-xylazine (100 and 10 mg/kg, respectively) and intranasally infected with 10^4^ p.f.u. 2009 H1N1 (A/Hamburg/NY1580/09) influenza virus (11) diluted in 50 μl 1x phosphate buffered saline (PBS). Control groups received 50 μl 1x PBS. Animal numbers are provided in the legends. After infection, body weight and mortality were monitored for 14 days. At selected time points, whole lungs and plasma were collected for terminal studies.

### Surgical Procedures, Testosterone Administration, and Quantification

Male and female mice were anesthetized with 2.5% isoflurane mixed with oxygen. For gonadectomy, 6 weeks old male mice were assigned to remain intact or be bilaterally gonadectomized. Six weeks old female mice were subcutaneously implanted an ALZet Model 2004 micro-osmotic pump (Charles River) releasing either a carrier substance for the control group or testosterone [5 mg/ml diluted in 45% w/w (2-Hydroxypropyl)-ß-cyclodextrin]. One hour before and 24 h after surgery mice were administered subcutaneously with carprofen purchased from Zoetis (5 mg/kg) as postoperative analgetic. All infections occurred 2 weeks following the surgeries. Plasma testosterone concentrations were determined by a chemiluminescence immunoassay (ADVIA Centaur Testosterone II assays; Siemens Healthcare Diagnostics) and the measurements were performed with the ADVIA Centaur XP (Siemens Healthcare Diagnostics).

### Lung Pathology

Formalin-fixed paraffin-embedded lung thin sections were stained with hematoxylin a rabbit anti-NP antibody (Thermo Fisher, PA5-32242) and a biotin-conjugated-rabbit secondary antibody (Jackson ImmunoResearch, 711-066-152) for immunohistochemical analysis. All images were taken at 400x magnification with a wide-field microscope (Nikon Eclipse 80i live microscope).

### Pulmonary Cytokine/Chemokine Quantification and Analysis of Viral Lung Titers

Homogenization of ∼ 50 mg of lungs was performed in 1 ml PBS with 5 sterile, stainless steel beads (Ø 2 mm, #22.455.0010, Retch) at 30 Hz and 4°C for 10 min in the mixer mill MM400 (Retsch). Viral lung titers were determined by plaque assay as described previously (7). Cytokine and chemokine levels were analyzed using ProcartaPlex^TM^ multiplex immunoassays (Thermo Fisher) according to the manufacturer’s protocol. The following cytokines and chemokines were analyzed: interleukins (IL): IL-1β, IL-6, IL-10, IL-17A, interferon (IFN) α, tumor necrosis factor (TNF) α, and monocyte chemotactic protein (MCP) 1. The signal intensities were measured using Bio-Plex^®^ 200 Systems (Bio-Rad).

### Analysis of Sex Hormone Receptor Level Expression by RT-qPCR

Total RNA from PBMCs was isolated following a guanidinium thiocyanate-phenol-chloroform extraction protocol. The samples were diluted in TRIzol^®^ and treated with chloroform before centrifugation. After phase-separation, the precipitation of RNA was performed using isopropanol following a washing step with 75% Ethanol. The RNA was eluted in RNase-free water. RNA concentration and purity were determined using the Nanodrop 1000 (Peqlab). Total cDNA was generated using random nonamer primers (Gene Link^TM^, pd(N)9, 26-4000-06) and the SuperScript^TM^ III Reverse Transcriptase (Invitrogen) according to the manufacturer’s instructions. For the qPCR, specific primer pairs were used for the genes of interest (GOIs), murine estrogen receptor alpha (ESR1), murine androgen receptor (AR) and for the reference gene murine ribosomal protein 9 (Rsp9). The reactions were set up in MicroAmp^®^ Optical 96-Well Reaction Plates (Invitrogen) including Platinum^®^ SYBR^®^ Green qPCR SuperMix-UDG (ROCHE^®^), forward and reverse primer and the cDNA template. The RT-qPCRs were conducted on the LightCycler^®^ 96 Real-Time PCR System (ROCHE^®^) as described previously (12). The relative quantifications were performed using the −2^−ΔΔ*Ct*^ method.

The following primer sequences were used for qRT–PCR:

*murine ESR1* forward 5′-AGTGAAGCCTCAATGATGGG-3′,

reverse 5′-GAGCAAGTTAGGAGCAAACAG-3′,

*murine AR* forward 5′- TGAGTACCGCATGCACAAGT-3′,

reverse 5′- GCCCATCCACTGGAATAATGC-3′

### Data Analysis

All data were analyzed with the Prism software (GraphPad, 5.03) using Mantel–Cox test or Student’s *t*-test as indicated in the respective legends. Association between sex hormones and cytokines were determined using linear regression and correlation analysis (Pearson). Statistical significance was defined as *p* < 0.05 (^∗^*p* < 0.05, ^∗∗^*p* < 0.01, ^∗∗∗^*p* < 0.001).

## Results

### Testosterone Treatment Protects Female Mice From Lethal 2009 H1N1 Influenza A Virus Infection

Influenza A virus pathogenesis may vary depending on sex (2, 7). Here, we studied the impact of testosterone on influenza disease outcome in female and male mice.

**FIGURE 1:**
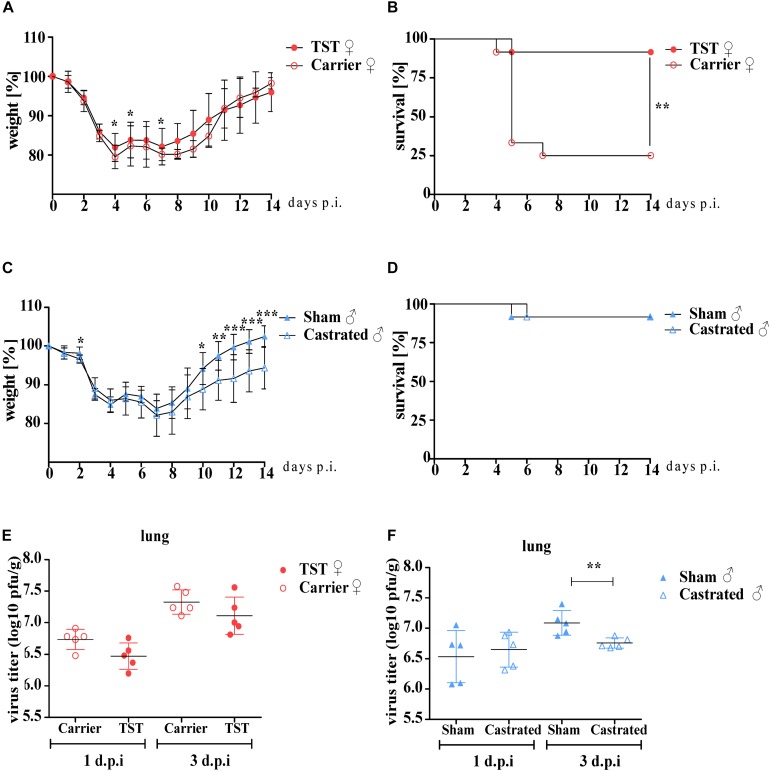
Testosterone impact on 2009 H1N1 influenza A virus pathogenicity in female and male C57BL/6 mice. Male mice (*n* = 12 each) were gonadectomized or sham-operated. Female mice (*n* = 12 each) were implanted an osmotic pump releasing either testosterone (TST) or a carrier substance. Female and male mice were intranasally infected with 1 × 10^4^ of the 2009 H1N1 influenza A virus. Weight loss and survival **(A–D)** were monitored for 14 days. Mean values and SD were determined. Statistical significance was assessed by Mantel–Cox test for the survival data and Student’s *t*-test for the weight loss data (*p < 0.05, **p < 0.01, ***p < 0.001). Lungs of five animals per group were harvested on days 1 and 3 d.p.i. Viral lung titers were determined by plaque assay **(E,F)**. The individual logarithmic virus titers of each lung and their means are shown. Statistical significance was assessed by Student’s *t*-test (*p < 0.05).

Female mice were either implanted with a testosterone releasing osmotic pump or with a carrier substance releasing pump as a negative control. Two weeks after surgery, testosterone and carrier treated female mice were infected with 2009 H1N1 influenza A virus (pH1N1). Testosterone treated females underwent reduced weight loss compared to carrier treated females (Figure 1A). While 2009 H1N1 infection was highly lethal (75%) in carrier treated females, testosterone treated females displayed high survival rates (92%) (Figure 1B).

Male mice were castrated or sham-operated to study the impact of testosterone on influenza disease outcome. Castrated male mice underwent more weight loss during the recovery phase compared to sham-operated control males upon 2009 H1N1 infection (Figure 1C). However, survival rates did not differ between infected castrated and non-castrated males (Figure 1D). Even increasing 2009 H1N1 infection dose did not significantly affect weight loss or survival rates (Supplementary Figure S1).

Virus replication titers in the lungs of testosterone treated female mice were comparable to carrier treated control females, albeit a tendency toward lower replication upon testosterone treatment could be detected at both 1 day and 3 days post infection (p.i.) (Figure 1E). Virus replication did not differ between castrated and sham-operated male mice on day 1 p.i. On day 3 p.i., virus replication was reduced in the lungs of castrated compared to control males (Figure 1F).

These findings show that female mice treated with testosterone are protected against lethal 2009 H1N1 influenza. However, improved survival rates do not correlate with reduced virus lung titers suggesting that the underlying mechanism is not primarily dependent on virus replication. Moreover, the protective role of testosterone is not observed in male mice.

### Testosterone Expression Levels Are Reduced in 2009 H1N1 Influenza A Virus Infected Male Mice

**FIGURE 2:**
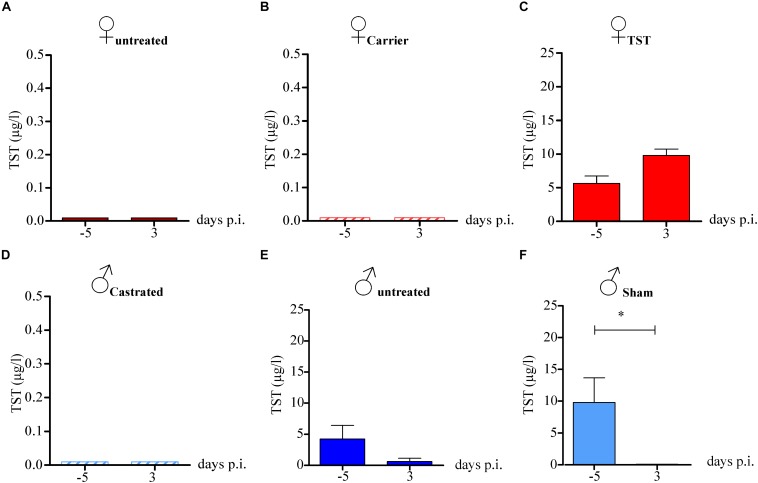
Testosterone levels in 2009 H1N1 influenza A virus infected female and male mice. Non-treated female mice **(A)**, female mice with an implanted osmotic pump releasing either a carrier substance **(B)** or testosterone (TST) **(C)** as well as gonadectomized **(D)** (*n* = 5 each), non-treated **(E)** and sham-operated male mice **(F)** (*n* = 10 each) were intranasally infected with 1 × 10^4^ of the 2009 H1N1 influenza A virus. Serum testosterone levels were measured on day 5 before infection and day 3 post infection. Statistical significance was assessed by Student’s *t*-test (*p < 0.05).

Next, we wanted to assess whether 2009 H1N1 infection might affect testosterone levels that in turn could affect disease outcome. Therefore, we measured testosterone levels in the plasma of mice 5 days (−5) before and 3 days (3) p.i. In female mice, either non-treated or treated with carrier substance, testosterone levels were below detection limits as expected (Figures 2A,B). Females treated with testosterone displayed testosterone levels within the physiological range of male mice before and during infection (Figure 2C). Castrated male mice did not show detectable testosterone levels as expected (Figure 2D). Male mice, either sham-operated or non-treated displayed high testosterone levels before infection. However, testosterone levels were significantly reduced in non-treated and sham-operated males on day 3 p.i. (Figures 2E,F). Then, we assessed whether changes in hormone levels might be due to altered hormone receptor expression. No significant differences in estrogen receptor (ESR1) and AR expression were detected in infected versus non-infected groups. However, a slight decrease ESR1 expression could be observed in testosterone treated female mice irrespective of infection (Supplementary Figure S2).

These findings show that 2009 H1N1 infection mediates reduced expression of testosterone in male mice. This observation may explain why influenza virus pathogenesis did not differ between castrated and non-castrated males.

### Testosterone Treatment Dampens Inflammatory IL-1β Response in 2009 H1N1 Influenza A Virus Infected Female Mice

**FIGURE 3:**
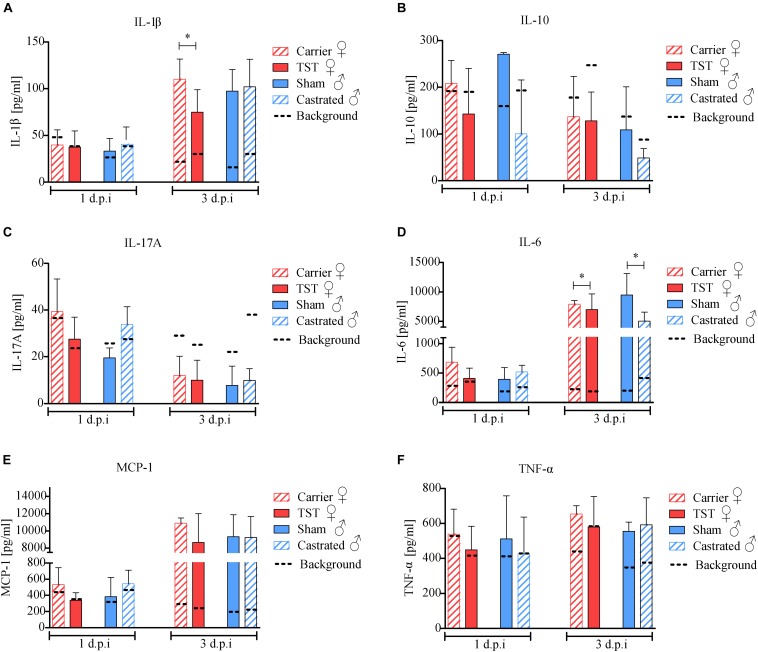
Testosterone impact on chemokine and cytokine responses in 2009 H1N1 influenza A virus infected female and male C57BL/6 mice. Gonadectomized or sham-operated male mice (*n* = 5 each) and female mice with an implanted osmotic pump releasing either testosterone (TST) or a carrier substance (*n* = 5 each) were intranasally infected with 1 × 10^4^ of the 2009 H1N1 influenza A virus or PBS. Lungs of five animals per group were harvested on days 1 and 3 d.p.i. Cytokines IL-1β **(A)**, IL-10 **(B)**, IL-17A **(C)**, IL-6 **(D)**, MCP-1 **(E)**, TNF-α **(F)** were determined in lung homogenate supernatants (*n* = 5) by a procartaplex cytokine multiplex assay. The individual mean values of the PBS control animals are indicated as dotted lines. Statistical significance was assessed by Student’s *t*-test (*p < 0.05).

Then, we addressed the question whether testosterone might affect pulmonary chemokine and cytokine responses in 2009 H1N1 infected mice thereby affecting disease outcome. As a control, cytokine and chemokine levels of PBS-treated mice were defined as background references and compared individually to the respective treated groups. Upon 2009 H1N1 infection, IL-1β, IL-6, and MCP-1 level were increased in all animals compared to their respective background references unlike IL-10, IL-17A, and TNF-α levels (Figures 3A–F). Testosterone treated female mice showed significantly decreased IL-1β levels compared to carrier substance treated females (Figure 3A). IL-6 level were slightly decreased in testosterone treated females and in castrated males (Figure 3D). However, cytokine and chemokine levels were not significantly altered in the respective male groups in line with similar disease outcomes.

These data show that testosterone treatment results in significantly reduced pro-inflammatory IL-1β expression in the lungs of 2009 H1N1 infected female mice correlating with elevated survival rates in females.

### Testosterone Reduces Lung Pathology in 2009 H1N1 Influenza A Virus Infected Mice

**FIGURE 4:**
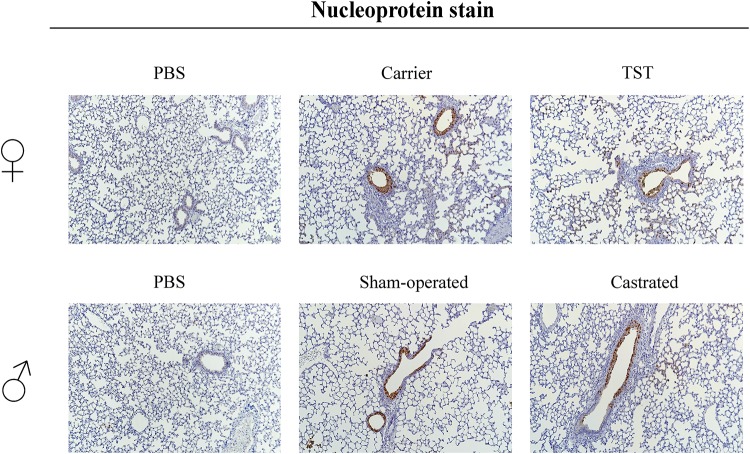
Testosterone impact on lung tropism of 2009 H1N1 influenza A virus infected mice. Female mice with an implanted osmotic pump releasing either a carrier substance or testosterone (TST), sham-operated or gonadectomized male mice (*n* = 5 each) were either intranasally infected with 1 × 10^4^ of the 2009 H1N1 influenza A virus or inoculated with PBS as a control. On day 3 p.i., lungs were harvested for histopathological analysis. Stainings were performed against influenza A virus nucleoprotein.

We then assessed the impact of testosterone treatment on lung pathology in 2009 H1N1 infected female and male mice. 2009 H1N1 infection resulted in antigen-positive bronchial as well as alveolar epithelium in female and male mice (Figure 4). Female mice treated with testosterone displayed reduced infiltration with mononuclear cells accompanied by less alveolar destruction compared to lungs of carrier-treated mice. Sham-operated and castrated male mice showed similar infiltration and virus positive cells.

These data show that testosterone treatment reduces lung pathology in female mice correlating with ameliorated influenza disease outcome.

## Discussion

In this study, we sought evidence for the epidemiological observation why men undergo less severe influenza than women. These clinical findings can be mimicked in murine infection models as shown earlier allowing now causal assessments (1, 7). In this study, we provide evidence that testosterone has a protective role in 2009 H1N1 disease outcome in females. Testosterone is known to have an anti-inflammatory impact (13), whereas estrogens promote inflammation (14, 15). Severe inflammation is associated with cytokine storm leading to severe influenza (16). However, this protective impact of testosterone is not observed in male mice. Detailed analysis of testosterone expression kinetics in male mice revealed that 2009 H1N1 influenza virus infection reduces testosterone expression levels in male mice. This phenomenon is not expressed in female mice likely due the generally low testosterone levels in females. Others have reported a decline of testosterone levels in young and elderly male mice before (10). However, the underlying mechanism how influenza A virus infection might dysregulate testosterone expression is unknown. It was reported before that cytokines might interfere with testosterone synthesis, albeit the detailed mechanism is still unclear (17). In this study, we further analyzed androgen and receptor expression levels to identify the potential target pathways. However, influenza A virus infection did not significantly alter androgen or ESR1 levels, respectively (Supplementary Figure S2). Thus, future studies are required to understand the viral interference with testosterone expression. However, we could identify that testosterone treatment of female mice improves virus induced lung damage without affecting respiratory virus replication kinetics. Improved disease outcome in influenza virus infected female mice upon testosterone treatment strongly correlated with reduced pro-inflammatory IL-1β cytokine levels in the lungs and high survival rates. IL-1β plays a key role in influenza virus mediated lung pathologies (18). Interestingly, it was shown before that aged male mice treated with testosterone had reduced pulmonary IL-1β levels compared to aged male mice with low testosterone levels after infection (19). Therefore, a potential correlation between testosterone and IL-1β serum level in female mice after infection has been analyzed (Supplementary Figure S3). The results indicate a trend for a negative correlation between testosterone levels and IL-1β levels. In summary, our data show that testosterone treatment of females significantly improves influenza disease outcome by dampening IL-1β responses and reducing virus induced lung damage. These findings highlight potential sex differences should be taken into consideration in developing new antiviral strategies against pandemic influenza.

## Data Availability Statement

All datasets generated for this study are included in the article/Supplementary Material.

## Ethics Statement

The animal study was reviewed and approved by the German authorities (Behörde für Gesundheit und Verbraucherschutz, Hamburg, Germany, license number 01/15) and conducted according to the FELASA guidelines of animal welfare. All animal experiments were performed according to the guidelines of the German Animal Welfare Regulation.

## Author Contributions

BT, SS-B, and GG designed the study. BT and SS-B performed the experiments, analyzed the data, and performed all animal infection experiments. BT and GG wrote the manuscript. JS, SH, and HL performed the mouse surgeries. SB, AP, and TB supported animal infection experiments. NK performed the lung histopathological analysis. TR measured and analyzed the testosterone levels in mice. All authors revised the manuscript.

## Conflict of Interest

The authors declare that the research was conducted in the absence of any commercial or financial relationships that could be construed as a potential conflict of interest.

## References

[1] Klein SL Hodgson A Robinson DP. Mechanisms of sex disparities in influenza pathogenesis. *J Leukoc Biol.* (2012) 92:67–73. 10.1189/jlb.0811427 22131346 PMC4046247

[2] WHO. *Sex, Gender and Influenza.* (2010). Available online at: https://www.who.int/gender-equity-rights/knowledge/9789241500111/en/ (accessed July 4, 2020).

[3] Jensen-Fangel S Mohey R Johnsen SP Andersen PL Sorensen HT Ostergaard L. Gender differences in hospitalization rates for respiratory tract infections in Danish youth. *Scand J Infect Dis.* (2004) 36:31–6. 10.1080/00365540310017618 15000556

[4] Eshima N Tokumaru O Hara S Bacal K Korematsu S Tabata M Sex- and age-related differences in morbidity rates of 2009 pandemic influenza a H1N1 virus of swine origin in japan. *PLoS One.* (2011) 6:e19409. 10.1371/journal.pone.0019409 21559366 PMC3084848

[5] Jacobs JH Archer BN Baker MG Cowling BJ Heffernan RT Mercer G Searching for sharp drops in the incidence of pandemic A/H1N1 influenza by single year of age. *PLoS One.* (2012) 7:e42328. 10.1371/journal.pone.0042328 22876316 PMC3410923

[6] Kumar A Zarychanski R Pinto R Cook DJ Marshall J Lacroix J Critically ill patients with 2009 influenza A(H1N1) infection in Canada. *JAMA.* (2009) 302:1872–9. 10.1001/jama.2009.1496 19822627

[7] Hoffmann J Otte A Thiele S Lotter H Shu Y Gabriel G. Sex differences in H7N9 influenza A virus pathogenesis. *Vaccine.* (2015) 33:6949–54. 10.1016/j.vaccine.2015.08.044 26319064

[8] WHO. Update on human cases of influenza at the human - animal interface. *Wkly Epidemiol Rec.* (2012) 88:137–44. 23586138

[9] Hall OJ Limjunyawong N Vermillion MS Robinson DP Wohlgemuth N Pekosz A Progesterone-based therapy protects against influenza by promoting lung repair and recovery in females. *PLoS Pathog.* (2016) 12:e1005840. 10.1371/journal.ppat.1005840 27631986 PMC5025002

[10] Robinson DP Hall OJ Nilles TL Bream JH Klein SL. 17beta-estradiol protects females against influenza by recruiting neutrophils and increasing virus-specific CD8 T cell responses in the lungs. *J Virol.* (2014) 88:4711–20. 10.1128/JVI.02081-13 24522912 PMC3993800

[11] Otte A Gabriel G. pandemic H1N1 influenza A virus strains display differential pathogenicity in C57BL/6J but not BALB/c mice. *Virulence.* (2009) 2:563–6. 10.4161/viru.2.6.18148 22030859

[12] Stanelle-Bertram S Walendy-Gnirss K Speiseder T Thiele S Asante IA Dreier C Male offspring born to mildly ZIKV-infected mice are at risk of developing neurocognitive disorders in adulthood. *Nat Microbiol.* (2018) 3:1161–74. 10.1038/s41564-018-0236-1 30202017

[13] Gilliver SC. Sex steroids as inflammatory regulators. *J Steroid Biochem Mol Biol.* (2010) 120:105–15. 10.1016/j.jsbmb.2009.12.015 20045727

[14] Calippe B Douin-Echinard V Delpy L Laffargue M Lélu K Krust A 17β-Estradiol promotes TLR4-triggered proinflammatory mediator production through direct estrogen receptor α signaling in macrophages in vivo. *J Immunol.* (2010) 185:1169–76. 10.4049/jimmunol.0902383 20554954

[15] Pratap UP Sharma HR Mohanty A Kale P Gopinath S Hima L Estrogen upregulates inflammatory signals through NF-κB, IFN-γ, and nitric oxide via Akt/mTOR pathway in the lymph node lymphocytes of middle-aged female rats. *Int Immunopharmacol.* (2015) 29:591–8. 10.1016/j.intimp.2015.09.024 26440402

[16] Tisoncik JR Korth MJ Simmons CP Farrar J Martin TR Katze MG. Into the eye of the cytokine storm. *Microbiol Mol Biol Rev.* (2012) 76:16–32. 10.1128/mmbr.05015-11 22390970 PMC3294426

[17] Bornstein SR Rutkowski H Vrezas I. Cytokines and steroidogenesis. *Mol Cell Endocrinol.* (2004) 215:135–41. 10.1016/j.mce.2003.11.022 15026186

[18] Indalao IL Sawabuchi T Takahashi E Kido HIL-. 1beta is a key cytokine that induces trypsin upregulation in the influenza virus-cytokine-trypsin cycle. *Arch Virol.* (2017) 162:201–11. 10.1007/s00705-016-3093-3 27714503 PMC5225228

[19] vom Steeg LG Attreed SE Zirkin B Klein SL. Testosterone of aged male mice improves some but not all aspects of age-associated increases in influenza severity. *Cell Immunol.* (2019) 345:103988. 10.1016/j.cellimm.2019.103988 31540670 PMC6876866

