# Supplementary material for: Testosterone Protects Against Severe Influenza by Reducing the Pro-Inflammatory Cytokine Response in the Murine Lung
Source: Front Immunol. 2020 Apr 22;11:697. doi: 10.3389/fimmu.2020.00697 (PMC7216738; doi:10.3389/fimmu.2020.00697)
Supplement: Supplementary file 1 [file Data_Sheet_1.PDF]

Supplementary Figure 1

A

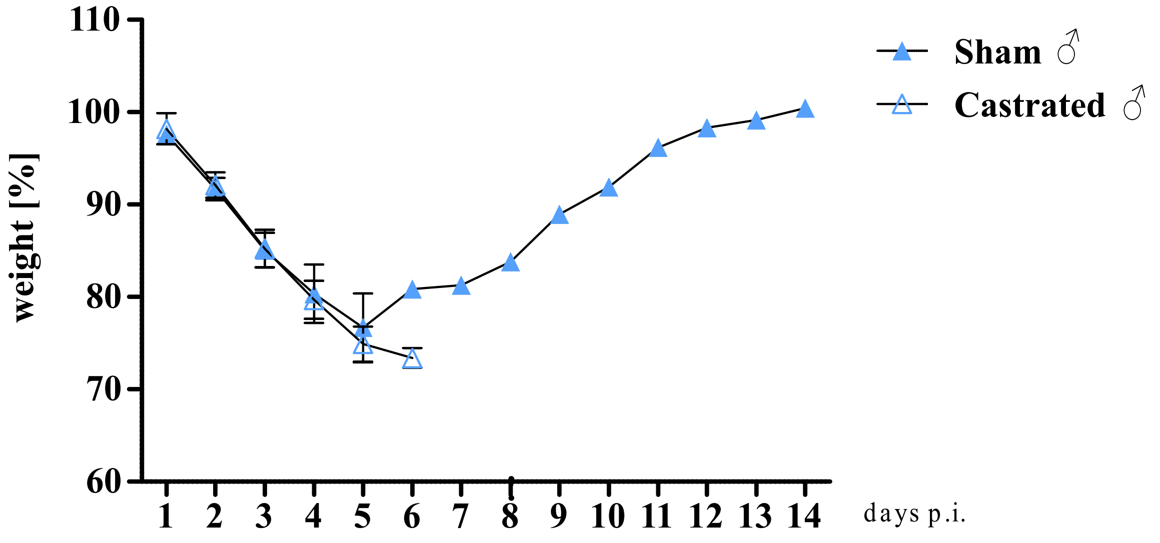

B

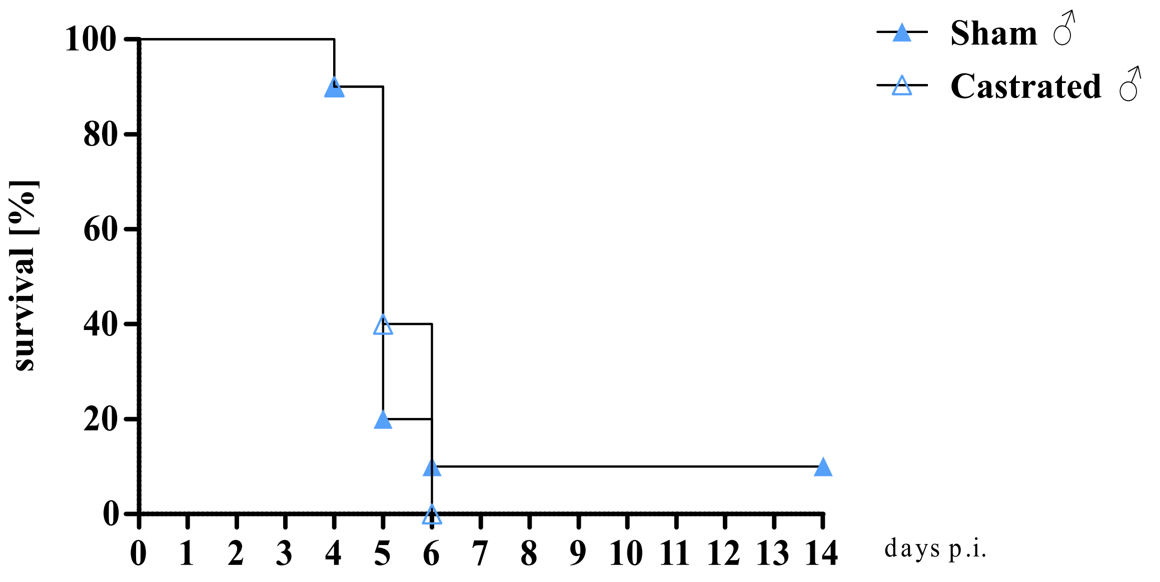

Supplementary Figure 1

Testosterone impact on high dose pathogenicity of 2009 pH1N1 influenza A virus in male C57BL/6 mice. Male mice (*n* = 10 each) were gonadectomized or sham-operated. Mice were intranasally infected with 1x 10^5 of the 2009 H1N1 influenza A virus. Weight loss (A) and survival (B) were monitored for 14 days. Mean values and SD were determined.

Supplementary Figure 2

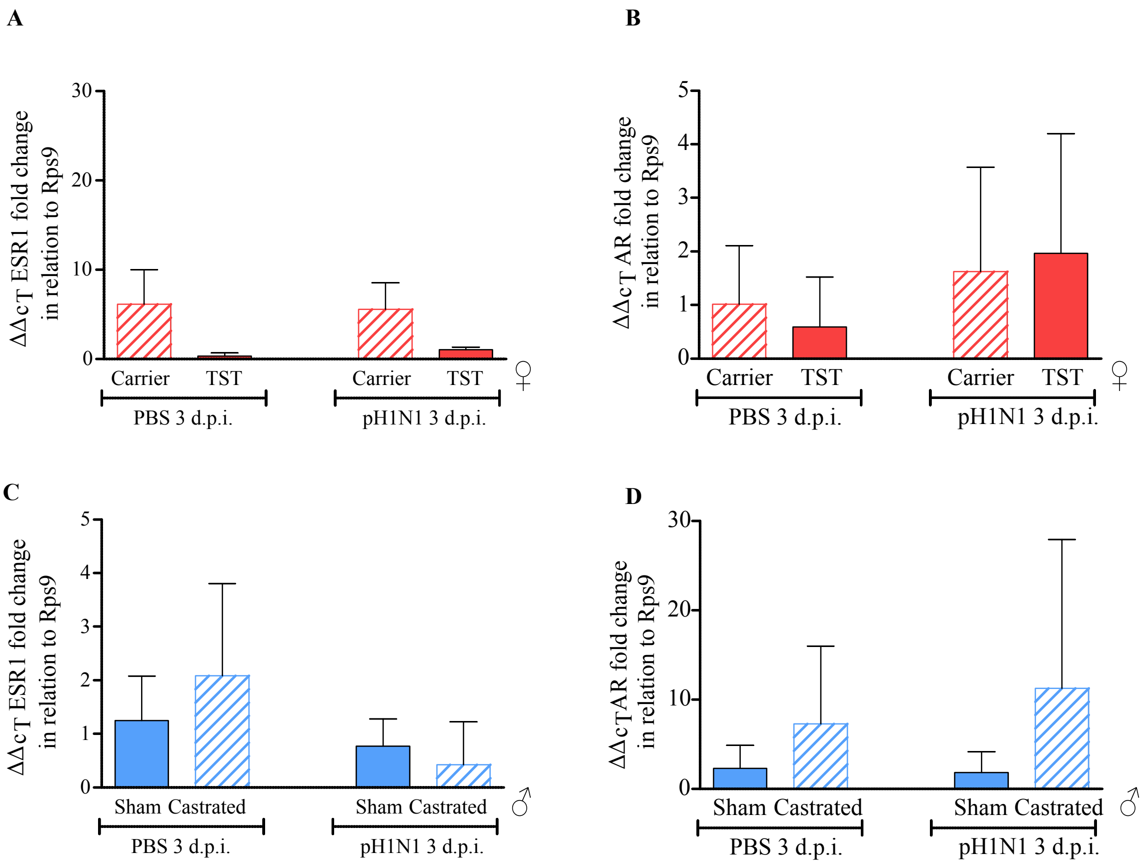

!

Supplementary Figure 2

Estrogen and androgen receptor expression levels in peripheral blood mononuclear cells (PBMCs).

Female mice with an implanted osmotic pump releasing either testosterone (TST) or a carrier substance (A-B) (n = 5 each) and gonadectomized or sham-operated male mice (C-D) (n = 5 each) were intranasally infected with 1x 10^4 of the 2009 H1N1 influenza A virus. Blood of five animals per group were harvested on day 3 d.p.i. After RNA extraction of isolated PBMCs and subsequent cDNA synthesis quantitative RT-PCRs were performed to measure the mRNA expression levels of androgen and estrogen receptors. Rsps9 was set as reference. Statistical significance was assessed by Mann-Whitney test.

Supplementary Figure 3

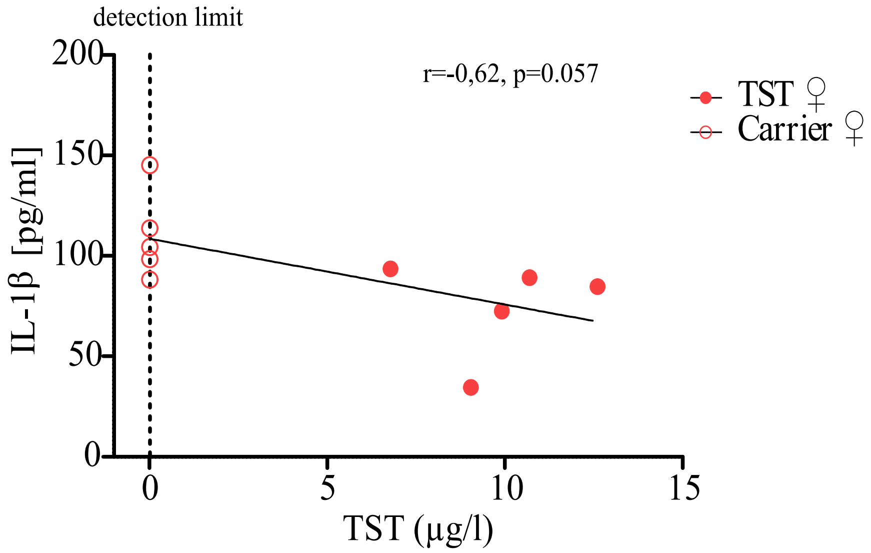

Supplementary Figure 3

Association between testosterone levels and IL-1β serum levels in female mice upon 2009 H1N1 infection

Female mice with an implanted osmotic pump releasing either testosterone (TST) or a carrier substance (*n* = 5 each) were intranasally infected with 1x 10^4 of the 2009 H1N1 influenza A virus. Blood of five animals per group were harvested on day 3 d.p.i. Serum testosterone levels as well as cytokine expression were determined using linear regression and correlational analysis (Pearson). The dotted line indicates the detection limit, whereas the testosterone levels of carrier female mice were set as 0,01.
